# Associations Between Poor Sleep Quality, Anxiety Symptoms, and Depressive Symptoms Among Chinese Adolescents Before and During COVID-19: A Longitudinal Study

**DOI:** 10.3389/fpsyt.2021.786640

**Published:** 2022-01-14

**Authors:** Wanxin Wang, Yangfeng Guo, Xueying Du, Wenyan Li, Ruipeng Wu, Lan Guo, Ciyong Lu

**Affiliations:** ^1^Department of Medical Statistics and Epidemiology, School of Public Health, Sun Yat-sen University, Guangzhou, China; ^2^Guangdong Provincial Key Laboratory of Food, Nutrition and Health, Sun Yat-sen University, Guangzhou, China; ^3^Department of Prevention and Treatment of Common Diseases Among Students, Health Promotion Center for Primary and Secondary Schools, Guangzhou, China

**Keywords:** mental health, adolescent, longitudinal study, COVID-19, sleep problems

## Abstract

Since the novel coronavirus disease 2019 (COVID-19) outbreak, adolescents' emerging mental health and behavior issues have been an international public health concern. This longitudinal study aimed to examine the situation of poor sleep quality, anxiety, and depressive symptoms among Chinese adolescents and to explore the associations between them before and during COVID-19. A total of 1,952 middle and high school students as eligible participants at baseline (pre-COVID-19, Wave 1; response rate: 98.79%), 1,831 eligible students were followed up at Wave 2 (October 2019 to December 2019, pre-COVID-19; retention rate: 93.80%), and 1,790 completed the follow-up at Wave 3 (during the COVID-19; retention rate: 97.80%). The mean age of the baseline students was 13.56 (SD: 1.46) years. The differences in anxiety and depressive symptoms between Wave 1, Wave 2, and Wave 3 were not statistically significant. The proportion of students with poor sleep quality increased over time, from Wave 1 (21.0%) to Wave 3 (26.0%, OR = 1.37, 95% CI = 1.17–1.60, *P* = 0.001) and from Wave 2 (21.9%) to Wave 3 (OR = 1.29, 95% CI = 1.11–1.51, *P* < 0.001). The cross-lagged generalized linear mixed models revealed that the concurrent and cross-lagged associations of poor sleep quality with anxiety symptoms across the three waves were significant (*P* < 0.05) and vice versa. Only a marginally significant positive cross-lagged association between poor sleep quality at Wave 2 and depressive symptoms at Wave 3 was found (standardized β estimate = 0.044, SE = 0.022, *P* = 0.045). Sleep quality was adversely affected during COVID-19, and the bidirectional associations of poor sleep quality with anxiety symptoms could not be neglected.

## Introduction

Adolescence represents a developmental transition period between childhood and adulthood, characterized by marked changes in biological systems and physical maturation of the body and brain, rendering adolescents vulnerable to mental health and behavior problems, including anxiety symptoms, depressive symptoms, and poor sleep quality (1). Anxiety and depressive symptoms are among the most prevalent mental health problems presented in adolescents, resulting in considerable distress to the individual (e.g., impaired school functioning or developing into mental disorders), and then are the main contributors to the global burden of disease in this population (2). However, the causes of anxiety symptoms and depressive symptoms remained unclear. Previous evidence has suggested that sleep is an important predictor of adolescents' subsequent anxiety and depressive problems (3). Sleep is a core behavior of adolescents, consuming up to a third or more of each day, and contributes a vital role in adolescent brain development (4). With the increased incidence and prevalence of poor sleep quality reported in adolescents from different countries, adolescent sleep health is increasingly becoming a global public health concern. Numerous studies have found that poor sleep quality was associated with the elevated risks of anxiety symptoms or depressive symptoms (5, 6). Some evidence has also shown that anxiety symptoms or depressive symptoms can reversely predict poor sleep among adolescents (7), indicating that the associations of poor sleep quality with anxiety symptoms or depressive symptoms may be complex and bidirectional.

Since the outbreak of the novel coronavirus disease 2019 (COVID-19), the emerging mental health and behavior issues related to this global event have been an international public health concern (8). Previous cross-sectional and longitudinal studies in China during the initial stages of the COVID-19 pandemic demonstrated elevated levels of mental health problems among the general population (9, 10). The effects of the COVID-19 pandemic and the policies to contain it can be considered a stressor exposure, and their effects on mental health and behavioral problems are probably to be profound and long-lasting. However, to what extent such effects will last generalize to which population is unclear. During the early stages of the COVID-19 pandemic, most governments around the world have temporarily closed schools. As the outbreak of the COVID-19 during Chinese schools' winter vacation, all the school students were at home. It was notable that China's Ministry of Education issued school closure policies for the entire country between January 20 and February 8, and announced on January 27, 2020 that the 2020 spring semester for schools would be postponed to late April due to the COVID-19 outbreak, affecting a large number of students across primary and post-secondary grades in China (11). Some recent studies have discussed the influences of school closures during the COVID-19 pandemic on Chinese adolescents' anxiety or depressive symptoms and sleep behavior (12, 13). Nonetheless, most of the available evidence is cross-sectional or short-term and does not address the longer-term effects of COVID-19 and related policies on adolescents' mental health and behavior. Moreover, there is a scarce study on the longitudinal and reciprocal associations between poor sleep quality, anxiety symptoms, and depressive symptoms among Chinese adolescents before and during the COVID-19 pandemic. It is also notable that to minimize the negative impact of COVID-19 school closures on students, the government, National Health Commission, schools, families, and mental health professionals worked together to maintain students' routines and protect them from the impact of the pandemic (including guidelines, recommendations, or interventions during home confinement; online help services; a reduction in academic load) (14–16).

Therefore, longitudinal studies are needed to assess this gap in the literature. Cascade modeling, an analytic approach controlling within-time and across-time associations while estimating cross-lagged effects, is a strategy of examining the complex interplay in multiple factors across time (17). The purposes of this three-wave longitudinal study were to examine the situation of poor sleep quality, anxiety symptoms, and depressive symptoms among Chinese adolescents before and during COVID-19; to examine the concurrent and longitudinal associations among poor sleep quality, anxiety symptoms, and depressive symptoms among these adolescents at three-time points.

## Materials and Methods

### Study Design and Participants

Data were drawn from the Longitudinal Study of Adolescents' Mental and Behavioral Well-being Research in Guangzhou, China (Registration No. ChiCTR1900022032) (18), which used a multi-stage stratified cluster random sampling method to recruit 1,976 grade 7 and grade 10 students. Full details of the study sampling method are reported elsewhere (18). In brief, six middle schools and four high schools in Guangzhou were selected according to the representative and proportion of middle school and high school across districts, convenience for the data collection, and previous study collaboration. Two classes from the 7^th^ grade in middle school and the 10^th^ grade in high school were randomly selected among the chosen schools. All students presenting in the selected classes were invited to participate voluntarily. In this study, a total of 1,952 students as eligible participants at baseline (January to March 2019, pre-COVID-19, Wave 1; response rate: 98.79%). Among the recruited 1,952 students, 1,831 students were followed up at Wave 2 (October 2019 to December 2019, pre-COVID-19; retention rate: 93.80%), and 1,790 completed the follow-up at Wave 3 (October 2020 to December 2020, during the COVID-19; retention rate: 97.80%). After almost 3 months of lockdown between Wave 2 and Wave 3, all the surveyed middle and high schools in Guangzhou were reopened on May 11. In order to protect the privacy of participants and reduce information bias, self-reported questionnaires were blind to teachers during the three waves. After the study had been fully explained in detail, written informed consent was obtained from each participant and one of the student's legal guardians. This study was approved by the Sun Yat-sen University, School of Public Health Institutional Review Board (Ethics Number: L2017060).

### Measures

#### Anxiety Symptoms

Anxiety symptoms were measured by the Chinese version of the Generalized Anxiety Disorder 7-Item Questionnaire (GAD-7) (19), which has been validated and widely used in Chinese adolescents with acceptable psychometric properties (20, 21). The Cronbach's alpha was 0.89, 0.91, and 0.91 with the study sample in Wave 1, Wave 2, and Wave3, respectively. The respondents were asked to rate the frequency of anxiety symptoms over the last 2 weeks, with the response options given on a four-point Likert scale (0 = not at all, 1 = several days, 2 = more than half the days, and 3 = nearly every day). The sum of scores ranges from 0 to 21, with higher scores indicating a higher level of anxiety symptoms severity, with the GAD-7 score ≥5 used to interpret as having anxiety symptoms (19).

#### Depressive Symptoms

Depressive symptoms were assessed by the Chinese version of the Center for Epidemiology Scale for Depression (CES-D), which has been validated and extensively utilized in Chinese adolescents (22, 23). The Cronbach's alpha was 0.80, 0.83, and 0.84 with the study sample in Wave 1, Wave 2, and Wave3, respectively. The CES-D comprises 20 items about the frequency of symptoms of depression over the past week, and the response options were rated in a four-point Likert scale (from 0 = rarely or none of the time to 3 = most or all of the time). The total CES-D score ranges from 0 to 60, with higher scores indicating more severe depressive symptomatology. A total CES-D score ≥16 was considered as having depressive symptoms (24).

#### Poor Sleep Quality

The level of sleep quality was assessed by the Chinese version of the Pittsburgh Sleep Quality Index (PSQI), which is commonly used to measure sleep quality and disturbance over 1 month. The Chinese version of PSQI has been validated and widely used among Chinese adolescents (25). The PSQI consists of 19 items constituting seven subscales: subjective sleep quality, sleep latency, sleep duration, habitual sleep efficiency, sleep disturbance, sleep medication use, and daytime dysfunction. The scores for each subscale are weighted equally and range from 0 to 3 points, and the sum of the scores for these seven subscales yields a global PSQI score, which ranges from 0 to 21, with higher scores indicating worse sleep quality. A global PSQI score >7 points indicates having poor sleep quality (25).

#### Other Variables

Demographic information including age, sex (1 = boy, 2 = girl), living arrangement (1 = living with both parents, 2 = living with a single parent, 3 = living with others), family relations (1 = good, 2 = average, 3 = poor), student-teacher relations (1 = good, 2 = average, 3 = poor), ever smoking (1 = yes, 2 = no), ever drinking (1 = yes, 2 = no) were also collected.

### Statistical Analysis

All statistical analyses were conducted using Stata/MP (Stata Corp, LLC, College Station, TX) and Mplus version 8.3 (Muthén & Muthén, Los Angeles, CA, USA). First, descriptive analyses were used to describe sample characteristics across three waves, and the Chi-square tests for categorical variables and the one-way ANOVA analyses for continuous variables were performed. Second, considering this study utilized a complex multi-stage sampling design, students were grouped into classes, generalized linear mixed models (GLMMs) in which classes were treated as clusters were constructed to test the changes in anxiety symptoms, depressive symptoms, and poor sleep quality over the three waves for the whole sample (26). Third, the unadjusted and adjusted GLMMs were also adopted to examine the association of poor sleep quality (main effects) with anxiety symptoms or depressive symptoms over the three waves, including examination of the poor sleep quality by time interaction. Fourth, the cross-lagged autoregressive GLMMs were further used to examine the concurrent and cross-lagged associations of poor sleep quality with anxiety symptoms and depressive symptoms, and the unadjusted and adjusted cross-lagged autoregressive GLMMs were conducted in multiple steps. The comparative fit index (CFI), the standardized root mean square residual (SRMR), and the root mean square error of approximation (RMSEA) were used to measure model fit; CFI values >0.90 and RMSEA values <0.08 suggest acceptable model fit (27). Missing data were eliminated in the GLMMs. All statistical tests were two-sided, and a *P* < 0.05 was considered statistically significant.

## Results

Table 1 shows sample demographic characteristics across waves. At wave 1, 50.7% of the participants were boys, and the mean (SD) age of the total students was 13.56 (SD: 1.46) years, with the age range from 13 to 18 years. The majority of students lived with both parents (81.4%), 3.0% reported poor family relations, and 1.4% reported their student-teacher relations as inferior. The proportion of students who reported ever smoking was 1.4% and reported ever drinking was 32.7%, respectively. There was no statistically significant difference in the demographic characteristics of the participants between the three waves (*P* > 0.05).

**Table 1:** Demographic characteristics of the participants across waves for all participants.

**Variable**	**Wave 1 (*n* = 1,952)**	**Wave 2 (*n* = 1,831)**	**Wave 3 (*n* = 1,790)**	***P*** value^*^
**Sex**				
Boys	989 (50.7)	918 (50.1)	899 (50.2)	0.945
Girls	963 (49.3)	913 (49.9)	880 (49.2)	
Missing data	0	0	11 (0.6)	
**Age, mean (SD), year**	13.56 (1.46)	14.04 (1.43)	14.92 (1.55)	
**Living arrangement**				
Living with both parents	1,589 (81.4)	1,503 (82.1)	1,484 (82.9)	0.258
Living with a single parent	192 (9.8)	192 (10.5)	185 (10.3)	
Living with others	167 (8.6)	135 (7.4)	119 (6.6)	
Missing data	4 (0.2)	1 (0.1)	2 (0.1)	
**Family relations**				
Good	1,655 (84.8)	1,512 (82.6)	1,478 (82.6)	0.180
Average	230 (11.8)	235 (12.8)	229 (12.8)	
Poor	59 (3.0)	77 (4.2)	74 (4.1)	
Missing data	8 (0.4)	7 (0.4)	9 (0.5)	
**Student-teacher relations**				
Good	1,601 (82.0)	1,480 (80.8)	1,442 (80.6)	0.347
Average	306 (15.8)	313 (17.1)	324 (18.1)	
Poor	27 (1.4)	27 (1.5)	20 (1.1)	
Missing data	18 (0.9)	11 (0.6)		
**Ever smoking a cigarette**				
Yes	28 (1.4)	34 (1.9)	45 (2.5)	0.053
No	1,914 (98.1)	1,793 (97.9)	1,735 (96.9)	
Missing data	10 (0.5)	4 (0.2)	10 (0.6)	
**Ever drinking alcohol**				
Yes	639 (32.7)	606 (33.1)	559 (31.2)	0.494
No	1,303 (66.8)	1,217 (66.5)	1,216 (67.9)	
Missing data	10 (0.5)	8 (0.4)	15 (0.8)	

* *The chi-square test was used for categorical variables and the one-way ANOVA analysis was used for age data*.

The mean GAD-7 score of anxiety symptoms at Wave 1, Wave 2, and Wave 3 was 3.75 (SD: 4.31), 3.60 (SD: 4.32), and 3.56 (SD: 4.22), respectively. Then mean CES-D score of depressive symptoms at Wave 1, Wave 2, and Wave 3 was 13.62 (SD: 10.07), 13.69 (SD: 10.53), and 13.44 (SD: 10.28), respectively. The differences in anxiety and depressive symptoms between Wave 1, Wave 2, and Wave 3 were not statistically significant. The mean PSQI score of sleep quality at Wave 1, Wave 2, and Wave 3 was 4.94 (SD: 2.44), 4.66 (SD: 2.84), and 5.08 (SD: 2.91), respectively; the difference between Wave 3 and Wave 2 was statistically significant (OR = 1.55, 95% CI 1.27–1.90, *P* < 0.001). Moreover, we also observed that compared with the sleep situation in Wave 1, there was a significant worsening of sleep duration (OR = 1.47, 95% CI = 1.35–1.60), sleep disturbance (OR = 2.11, 95% CI = 1.86–2.38), use of sleep medication (OR = 1.48, 95% CI = 1.10–1.99), daytime dysfunction (OR = 3.59, 95% CI = 3.22–4.00) in Wave 3, and a significant improvement in sleep latency (OR = 0.77, 95% CI = 0.71–0.84) in Wave 3. No significant differences were found between wave 1, wave 2, and wave 3 about subjective sleep quality and habitual sleep efficiency. The proportion of students with poor sleep quality increased over time, from Wave 1 (21.0%) to Wave 3 (26.0%, OR = 1.37, 95% CI = 1.17–1.60, *P* = 0.001) and from Wave 2 (21.9%) to Wave 3 (OR = 1.29, 95% CI = 1.11–1.51, *P* < 0.001), but not from Wave 1 to Wave 2 (OR = 1.05, 95% CI = 0.91–1.23, *P* = 0.471) (Table 2). Factors associated with anxiety symptoms, depressive symptoms, and poor sleep quality at Wave 1, Wave 2, and Wave 3 were presented in Supplementary Material.

**Table 2:** Changes in anxiety symptoms, depressive symptoms, and poor sleep quality over the three waves with odds ratios (ORs) and 95% confidence interval.

	**Wave 1 (*n* = 1,952)**	**Wave 2 (*n* = 1,831)**	**Wave 3 (*n* = 1,790)**	**OR (95% CI)**, ***P***		
				**Wave 2-Wave 1^**a**^**	**Wave 3-Wave 1^**a**^**	**Wave 3-Wave 2^**b**^**
GAD-7 scores, mean (SD)	3.75 (4.31)	3.60 (4.32)	3.56 (4.22)	0.86 (0.65~1.13), 0.269	0.82 (0.62~1.10), 0.184	0.96 (0.72~1.29), 0.797
Anxiety symptoms, yes, *n* (%)	637 (32.6)	579 (31.6)	589 (32.9)	1.02 (0.88~1.17), 0.803	0.95 (0.83~1.09), 0.507	1.08 (0.93~1.25), 0.312
CES-D scores, mean (SD)	13.62 (10.07)	13.69 (10.53)	13.44 (10.28)	1.07 (0.55~2.09), 0.837	0.84 (0.42~1.70), 0.633	0.78 (0.38~1.63), 0.518
Depressive symptoms, yes, *n* (%)	554 (28.4)	550 (30.0)	523 (29.2)	1.16 (0.94~1.44), 0.166	1.11 (0.89~1.39), 0.357	0.95 (0.76~1.19), 0.678
PSQI scores, mean (SD)	4.94 (2.44)	4.66 (2.84)	5.08 (2.91)	0.75 (0.63~0.90), 0.001	1.17 (0.97~1.41), 0.108	1.55 (1.27~1.90), <0.001
Subjective sleep quality, mean (SE)	0.99 (0.02)	1.00 (0.02)	1.00 (0.02)	1.03 (0.94~1.13), 0.573	1.02 (0.93~1.12), 0.690	0.99 (0.90~1.09), 0.884
Sleep latency, mean (SE)	0.74 (0.02)	0.73 (0.02)	0.70 (0.02)	0.98 (0.91~1.06), 0.608	0.77 (0.71~0.84), <0.001	0.79 (0.73~0.86), <0.001
Sleep duration, mean (SE)	0.48 (0.02)	0.56 (0.02)	0.72 (0.02)	1.15 (1.05~1.25), 0.002	1.47 (1.35~1.60), <0.001	1.28 (1.18~1.40), <0.001
Habitual sleep efficiency, mean (SE)	0.28 (0.02)	0.29 (0.02)	0.28 (0.01)	1.02 (0.92~1.13), 0.701	0.99 (0.90~1.10), 0.914	0.97 (0.88~1.08), 0.629
Sleep disturbance, mean (SE)	0.44 (0.01)	0.43 (0.01)	0.67 (0.01)	0.99 (0.87~1.12), 0.854	2.11 (1.86~2.38), <0.001	2.11 (1.86~2.40), <0.001
Sleep medication use, mean (SE)	0.02 (0.004)	0.03 (0.005)	0.04 (0.006)	1.20 (0.87~1.64), 0.270	1.48 (1.10~1.99), 0.009	1.25 (0.95~1.65), 0.109
Daytime dysfunction, mean (SE)	1.03 (0.01)	1.66 (0.02)	1.71 (0.02)	3.29 (2.96~3.65), 0.001	3.59 (3.22~4.00), <0.001	1.06 (0.98~1.14), 0.143
Poor sleep quality, yes, *n* (%)	409 (21.0)	401 (21.9)	466 (26.0)	1.05 (0.91~1.23), 0.471	1.37 (1.17~1.60), 0.001	1.29 (1.11~1.51), <0.001

*SD, standard deviation; SE, standard error; OR, odds ratio; 95% CI, 95% confidence interval*.

a *The reference group was wave 1*.

b *The reference group was wave 2*.

As shown in Table 3, the unadjusted and adjusted GLMMs revealed the absence of a significant interactive effect of poor sleep quality by time on anxiety symptoms (*P* > 0.05) and depressive symptoms (*P* > 0.05) through the three-wave follow-up. Moreover, without adjusting for other variables, the total PSQI sleep quality scores were positively associated with anxiety symptoms and depressive symptoms (*P* < 0.05).

**Table 3:** Prospective associations of poor sleep quality with anxiety symptoms and depressive symptoms.

	**Anxiety symptoms (GAD-7 scores)**				**Depressive symptoms (CES-D scores)**			
	**Model 1**		**Model 2**		**Model 1**		**Model 2**	
	**Unstandardized β**	***P***	**Unstandardized β**	***P***	**Unstandardized β**	***P***	**Unstandardized β**	***P***
	**estimate (95% CI)**		**estimate (95% CI)**		**estimate (95% CI)**		**estimate (95% CI)**	
**Time**								
Wave 1	1.00 (reference)		1.00 (reference)		1.00 (reference)		1.00 (reference)	
Wave 2	0.22 (−0.30~0.74)	0.626	0.24 (−0.27~0.75)	0.356	0.35 (−0.79~1.49)	0.550	0.13 (−0.97~1.23)	0.817
Wave 3	−0.14 (−0.68~0.41)	0.402	−0.04 (−0.59~0.50)	0.883	−0.08 (−1.28~1.13)	0.901	−0.24 (−1.44~0.96)	0.696
**Interaction**								
Poor sleep quality × Wave 1	1.00 (reference)		1.00 (reference)		1.00 (reference)		1.00 (reference)	
Poor sleep quality × Wave 2	−0.03 (−0.15~0.10)	0.684	−0.05 (−0.17~0.07)	0.410	0.09 (−0.18~0.35)	0.520	0.06 (-0.19~0.30)	0.641
Poor sleep quality × Wave 3	−0.03 (−0.15~0.09)	0.633	−0.03 (−0.15~0.09)	0.624	−0.05 (−0.31~0.21)	0.695	−0.05 (-0.29~0.20)	0.718
PSQI scores (1-score increase)	0.79 (0.69~0.89)	<0.001	0.69 (0.59~0.79)	<0.001	1.95 (1.75~2.16)	<0.001	1.61 (1.42~1.80)	<0.001
Subjective sleep quality (1-score increase)	1.14 (0.97~1.34)	0.120	1.17 (1.01–1.37)	0.043	1.22 (0.82–1.81)	0.328	1.32 (0.92–1.89)	0.130
Sleep latency (1-score increase)	0.99 (0.87~1.12)	0.842	1.04 (0.92–1.18)	0.498	0.98 (0.72–1.34)	0.895	1.14 (0.86–1.51)	0.364
Sleep duration (1-score increase)	1.06 (0.91–1.22)	0.458	1.09 (0.95–1.26)	0.205	0.92 (0.65–1.31)	0.650	1.00 (0.73–1.38)	0.994
Habitual sleep efficiency (1-score increase)	0.88 (0.73–1.05)	0.152	0.88 (0.74–1.04)	0.134	0.80 (0.52–1.23)	0.299	0.83 (0.56–1.23)	0.351
Sleep disturbance (1-score increase)	1.12 (0.90–1.38)	0.310	1.07 (0.88–1.31)	0.485	1.15 (0.69–1.92)	0.602	1.01 (0.64–1.62)	0.952
Sleep medication use (1-score increase)	1.16 (0.71–1.90)	0.555	0.98 (0.61–1.57)	0.919	1.37 (0.42–4.53)	0.601	1.12 (0.37–3.38)	0.839
Daytime dysfunction (1-score increase)	1.01 (0.88–1.16)	0.865	0.99 (0.87–1.13)	0.921	1.00 (0.72–1.40)	0.989	0.95 (0.70–1.29)	0.743

*Model 1: The unadjusted generalized linear mixed models*.

*Model 2: The generalized linear mixed models adjusted for age, sex, living arrangement, family relations, student-teacher relations, ever smoking, and ever drinking*.

After adjusting for age, sex, living arrangement, family relations, student-teacher relations, ever smoking, and ever drinking, there was a significant main effect of the total PSQI scores with anxiety symptoms (unstandardized β estimate = 0.69, 95% CI = 0.59~0.79, *P* < 0.001) and depressive symptoms (unstandardized β estimate = 1.61, 95% CI = 1.42~1.80, *P* < 0.001) through the three waves. Regarding each PSQI component, the adjusted GLMMs found that subjective sleep quality was positively associated with anxiety symptoms (unstandardized β estimate = 1.17, 95% CI = 1.01~1.37, *P* < 0.05) through the three waves.

**Figure 1:**
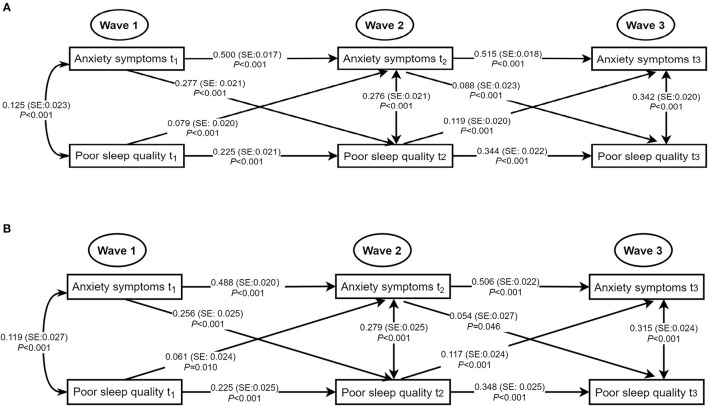
Concurrent and cross-lagged associations between poor sleep quality and anxiety symptoms. **(A)** Unadjusted models. **(B)** Models adjusting for age, sex, living arrangement, family relations, student-teacher relations, ever smoking, and ever drinking; for clarity of presentation, the standardized coefficients of the control variables were not displayed in the model but were included in the analyses. SE, standard error; the solid line meant the standardized coefficient was statistically significant.

To further observe the associations of poor sleep quality with anxiety symptoms and depressive symptoms, we examined cross-lagged GLMMs. Figure 1 depicted the results of the path models for poor sleep quality and anxiety symptoms with acceptable model fits that included all cross-lagged pathways. After adjusting for age, sex, living arrangement, family relations, student-teacher relations, ever smoking, and ever drinking, the adjusted autoregressive associations of both variables from Wave 1 to Wave 3 were positive and significant, indicating that baseline levels of poor sleep quality (standardized β estimate: ranging from 0.225 to 0.348) and anxiety symptoms (standardized β estimate: ranging from 0.488 to 0.506) were predictive of the same variable at follow-up. Moreover, the concurrent associations of poor sleep quality and anxiety symptoms at Wave 1, Wave 2, and Wave 3 were significant (at Wave 1: standardized β estimate = 0.119, *SE* = 0.027, *P* < 0.001; at Wave 2: standardized β estimate = 0.279, *SE* = 0.025, *P* < 0.001; at Wave 3: standardized β estimate = 0.315, *SE* = 0.024, *P* < 0.001). The significant positive cross-lagged associations between poor sleep quality (at Wave 1 or Wave 2) and subsequent anxiety symptoms (at Wave 2: standardized β estimate = 0.061, *SE* = 0.024, *P* = 0.010; at Wave 3: standardized β estimate = 0.117, *SE* = 0.024, *P* < 0.001). Conversely, high level of anxiety symptoms (at Wave 1 or Wave 2) was also associated with increased levels of poor sleep quality later (at Wave 2: standardized β estimate = 0.256, *SE* = 0.025, *P* < 0.001, *P* < 0.001; at Wave 3: standardized β estimate = 0.054, *SE* = 0.027, *P* = 0.046).

**Figure 2:**
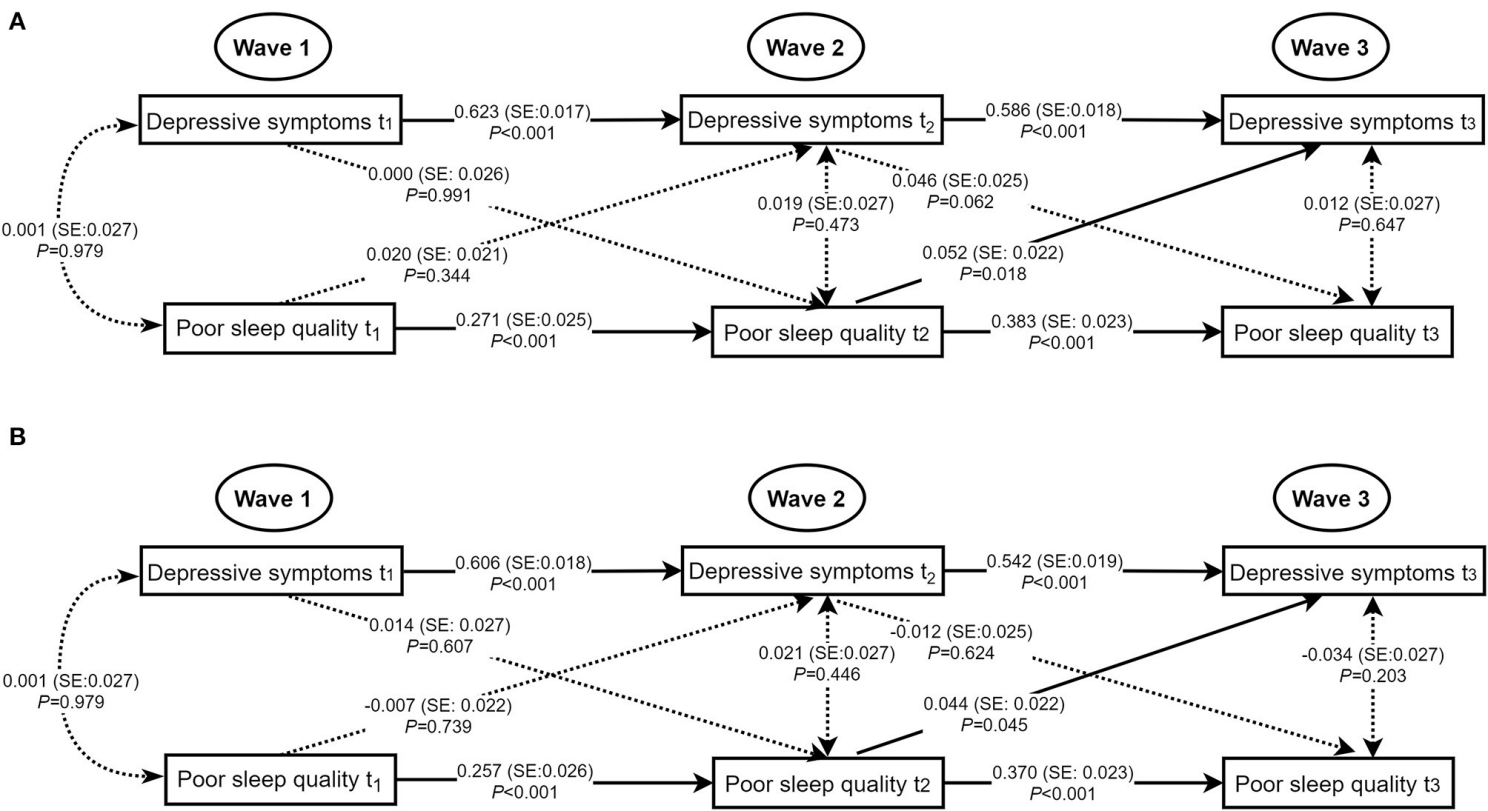
Concurrent and cross-lagged associations between poor sleep quality and depressive symptoms. **(A)** Unadjusted models. **(B)** Models adjusting for age, sex, living arrangement, family relations, student-teacher relations, ever smoking, and ever drinking; for clarity of presentation, the standardized coefficients of the control variables were not displayed in the model but were included in the analyses. SE, standard error; the solid line meant the standardized coefficient was statistically significant.

Figure 2 showed significantly positive autoregressive associations of poor sleep quality and depressive symptoms with the same variable from Wave 1 to Wave 3 in the unadjusted and adjusted models with acceptable model fits. After adjusting for age, sex, living arrangement, family relations, student-teacher relations, ever smoking, and ever drinking, no concurrent associations of poor sleep quality with depressive symptoms were statistically significant at Wave 1, Wave 2, and Wave 3. A marginally significant positive cross-lagged association between poor sleep quality at Wave 2 and depressive symptoms at Wave 3 was observed (standardized β estimate = 0.044, *SE* = 0.022, *P* = 0.045).

## Discussion

This study first provides a detailed examination of anxiety and depressive symptoms and poor sleep quality of the Chinese adolescents at three-time points, including the period before and during COVID-19. Although the found rate of anxiety symptoms, depressive symptoms, or poor sleep quality at Wave 3 in this study was similar to results from previous cross-sectional studies conducted during the COVID-19 (10, 12), our three-wave longitudinal study found that except for poor sleep quality, the situation of anxiety and depressive symptoms among adolescents did not get worse at Wave 3, which is the time point that students have experienced almost 3 months lockdown and school closure due to the COVID-19 pandemic. However, a previous longitudinal study showed that depressive symptoms (not anxiety symptoms) among Chinese primary school students in Wave 2 (2 weeks after school reopening, mid-May 2020) increased from levels at Wave 1 (before the outbreak of COVID-19) (28). One explanation of the differences of change in depressive symptoms might be that our study population were middle and high school students (not primary school students). Another possible explanation was that the assessing point of Wave 3 in our study was almost five months after school reopening, mainly focusing on the long-term effects of school closing and opening during the COVID-19 pandemic. Besides, it also should be noted that the government, schools, families, and mental health professionals worked together to maintain students' routines and protect them from the impact of the pandemic (14–16), and superior to usual preventive strategies have also been highly recommended to be implemented (e.g., school mental health consultation or mental health education) after school reopening. Consistent with our findings, a longitudinal study of adults in the UK demonstrated that anxiety and depressive symptoms remained stable across waves during the first 6 weeks of the COVID-19 pandemic (29); a recent longitudinal study among high school students in São Paulo also showed that adolescents delayed bed and wake-up times and had increased the global PSQI score during the COVID-19 pandemic (30). Moreover, our study also observed that regarding each component of PSQI, sleep duration, sleep disturbance, use of sleep medication, and daytime dysfunction got worse at Wave 3 among these adolescents, while sleep latency got better at Wave 3. These findings were partially similar to a previous study among adults (31), and suggested that families, schools, and clinicians should be informed about the adverse effects of the COVID-19 pandemic in relation to adolescent sleep quality.

Although the direct long-term influence of the COVID-19 pandemic on anxiety or depressive symptoms was not observed in this study, this study observed significant main longitudinal effects of poor sleep quality on anxiety symptoms and depressive symptoms through the three waves in with and without adjusted GLMMs. Moreover, prior studies also demonstrated that individuals with poor sleep quality were at a higher risk of depressive symptoms and anxiety symptoms than those without (32). Poor sleep quality is nearly universal in individuals with mental health problems, and previous evidence has suggested individuals with depressive or anxiety symptoms would have sleep quality complaints (33). Therefore, this study also investigated the bidirectional associations of poor sleep quality with anxiety and depressive symptoms. This study revealed that after adjusting for age, sex, living arrangement, family relations, student-teacher relations, ever smoking, and ever drinking, poor sleep quality was positively associated with current as well as subsequent anxiety symptoms and vice versa. Only a marginally significant cross-lagged association between poor sleep quality at Wave 2 and depressive symptoms at Wave 3 was observed. Similarly, Kelly et al. showed that there were significant reciprocal associations of children's sleep/wake problems with anxiety and depressive symptoms using three waves data spanning 5 years (34). Robert et al. using data from a two-wave cohort study found that short sleep duration at baseline increased the risk of subsequent anxiety disorder twofold, but the reverse association was not significant (35). Shanahan et al. using a study of youth ages between 9 and 16, found that sleep problems predicted an increase in generalized anxiety disorders but only predicted depression when anxiety symptoms were also present, and vice versa (36). These results might be related to that several myriad mechanisms for the association between sleep problems and anxiety/depressive symptoms have been posited, including genes, hormones, regulatory systems (e.g., the hypothalamic-pituitary-adrenal axis), and cognitive process (37, 38). Sleep problems are sometimes a symptom of anxiety or depression, and sufficient sleep may serve an important role in developing adolescent brain regions (e.g., the prefrontal lobe), which is responsible for emotional regulation and cognitive function (39). Conversely, anxiety or depressive symptoms are disabling conditions among adolescents that result in emotional suffering and poor sleep quality (40). In this study, only poor sleep quality at Wave 2 predicting depressive symptoms at Wave 3 may reflect students' sleep quality was adversely affected by the COVID-19 pandemic. A possibility for the unobserved concurrent or cross-lagged associations between poor sleep quality and depressive symptoms may be that the autoregressive association between previous depressive symptoms and subsequent depressive symptoms is too strong to cover the effects of poor sleep quality.

As already mentioned, whereas symptoms of anxiety and depression remained stable across the three waves, sleep quality was adversely affected, and the bidirectional associations between poor sleep quality and anxiety or depressive symptoms could not be neglected. Thereby, the implications of this study highlight that prevention or intervention strategies targeted at cultivating healthy sleep habits and improving sleep quality are helpful to develop good mental health among adolescents, particularly during COVID-19.

The findings also highlight the importance of regularly assessing adolescents' mental health status, especially in adolescents with poor sleep quality. Although this study extends exiting literature on the associations between poor sleep quality, anxiety symptoms, and depressive symptoms among Chinese adolescents before and during COVID-19, several limitations should also be considered. First, the school-based longitudinal study only included school students as the study sample and did not include adolescents absent from schools. Second, indicators of poor sleep quality, anxiety symptoms, and depressive symptoms were based on self-report, then this study only comments on these symptoms of mental health and behaviors rather than psychiatric disorders. Third, given that most Chinese middle school students or high school students will transfer to other schools after graduation, this longitudinal study only invited junior grade one and senior grade one students to avoid loss of follow-up due to students going on for further study.

In conclusion, this three-wave longitudinal study found that anxiety and depressive symptoms among Chinese adolescents remained stable before and during COVID-19, while sleep quality among adolescents gets worse at the time point when students have experienced almost 3 months of lockdown and school closure due to the COVID-19 pandemic. Moreover, the concurrent and cross-lagged associations between poor sleep quality and anxiety symptoms across the three waves (before and during COVID-19) were statistically significant and vice versa. In contrast, only the cross-lagged effect of poor sleep quality at Wave 2 on depressive symptoms at Wave 3 was marginally significant. Considering the COVID-19 pandemic has been a global public health event, these study findings addressed that a proper surveillance system should be established in China to regularly assess adolescent mental health and behaviors. Schools and families should also be aware of the adverse effects of the COVID-19 pandemic, and positive education and intervention programs that cultivate adolescents' healthy sleep habits and positive emotion regulation are highly recommended to be developed to decrease risks of anxiety and depressive symptoms, especially among those with the current poor sleep quality.

## Data Availability Statement

The raw data supporting the conclusions of this article will be made available by the authors, without undue reservation.

## Ethics Statement

The studies involving human participants were reviewed and approved by Sun Yat-sen University, School of Public Health Institutional Review Board. Written informed consent to participate in this study was provided by the participants' legal guardian/next of kin.

## Author Contributions

LG and WW: full access to all of the data in the study and take responsibility for the integrity of the data and the accuracy of the data analysis, drafting of the manuscript, and statistical analysis. LG, WW, and CL: concept, design, administrative, technical, or material support. YG, XD, WL, and RW: acquisition, analysis, or interpretation of data. LG, WW, YG, XD, CL, RW, and WL: drafting of the manuscript. LG, WW, YG, and CL: supervision. All authors finally approve the submission of this paper.

## Funding

This work was supported by the National Natural Science Foundation of China (Grant Nos. 81761128030 and 81903339) and Natural Science Foundation of Guangdong Province (Grant No. 2019A1515011091).

## Conflict of Interest

The authors declare that the research was conducted in the absence of any commercial or financial relationships that could be construed as a potential conflict of interest.

## Publisher's Note

All claims expressed in this article are solely those of the authors and do not necessarily represent those of their affiliated organizations, or those of the publisher, the editors and the reviewers. Any product that may be evaluated in this article, or claim that may be made by its manufacturer, is not guaranteed or endorsed by the publisher.
