# Supplementary material for: Associations Between Poor Sleep Quality, Anxiety Symptoms, and Depressive Symptoms Among Chinese Adolescents Before and During COVID-19: A Longitudinal Study
Source: Front Psychiatry. 2022 Jan 14;12:786640. doi: 10.3389/fpsyt.2021.786640 (PMC8795609; doi:10.3389/fpsyt.2021.786640)
Supplement: Supplementary file 1 [file Table_1.DOCX]

| **Supplementary Table 1. Factors associated with anxiety symptoms, depressive symptoms, and poor sleep quality at Wave 1.** | | | | | | |
| --- | --- | --- | --- | --- | --- | --- |
|  | **Wave 1 (n=1952)^*^** | | | | | |
|  | **Anxiety symptoms** | ***P*** | **Depressive symptoms** | ***P*** | **Poor sleep quality** | ***P*** |
|  | **Unstandardized *β* (95% *CI*)** |  | **Unstandardized *β* (95% *CI*)** |  | **Unstandardized *β* (95% *CI*)** |  |
| **Sex** (Ref: Girls) |  |  |  |  |  |  |
| Boys | -1.18 (-1.56~-0.80) | <0.001 | -2.99 (-3.89~-2.09) | <0.001 | -0.17 (-0.39~0.05) | 0.134 |
| **Age (1-year increase)** | 0.09 (-0.05~0.22) | 0.198 | 0.56 (0.25~0.88) | 0.001 | 0.23 (0.15~0.31) | <0.001 |
| **Living arrangement** (Ref: Living with both parents) |  |  |  |  |  |  |
| Living with a single parent | 1.02 (0.28~1.75) | 0.007 | 2.53 (0.84~4.23) | 0.003 | 0.54 (0.15~0.94) | 0.008 |
| Living with others | 0.77 (-0.04~1.58) | 0.064 | 2.79 (1.03~4.56) | 0.002 | 0.43 (-0.01~0.88) | 0.055 |
| **Family relations** (Ref: Good) |  |  |  |  |  |  |
| Average | 2.27 (1.53~3.00) | <0.001 | 6.88 (5.36~8.40) | <0.001 | 1.16 (0.78~1.54) | <0.001 |
| Poor | 5.67 (4.09~7.25) | <0.001 | 13.60 (10.06~17.14) | <0.001 | 2.29 (1.41~3.17) | <0.001 |
| **Student-teacher relations** (Ref: Good) |  |  |  |  |  |  |
| Average | 1.56 (-0.65~3.78) | 0.166 | 6.83 (5.43~8.23) | <0.001 | 1.04 (0.72~1.35) | <0.001 |
| Poor | 2.12 (1.51~2.73) | <0.001 | 6.48 (1.92~11.05) | 0.005 | 0.99 (-0.23~2.21) | 0.112 |
| **Ever smoking** (Ref: No) | 0.88 (-0.63~2.39) | 0.252 | 6.13 (1.53~10.72) | 0.009 | 0.91 (-0.41~2.22) | 0.177 |
| **Ever drinking** (Ref. No) | 1.32 (0.89~1.76) | <0.001 | 3.72 (2.68~4.76) | <0.001 | 0.81 (0.56~1.06) | <0.001 |

Abbreviations: 95% CI, 95% confidence interval; Ref., reference.

*: Unadjusted generalized linear mixed models.

| **Supplementary Table 2. Factors associated with anxiety symptoms, depressive symptoms, and poor sleep quality at Wave 2.** | | | | | | |
| --- | --- | --- | --- | --- | --- | --- |
|  | **Wave 2 (n=1831)^*^** | | | | | |
|  | **Anxiety symptoms** | ***P*** | **Depressive symptoms** | ***P*** | **Poor sleep quality** | ***P*** |
|  | **Unstandardized *β* (95% *CI*)** |  | **Unstandardized *β* (95% *CI*)** |  | **Unstandardized *β* (95% *CI*)** |  |
| **Sex** (Ref: Girls) |  |  |  |  |  |  |
| Boys | -1.17 (-1.56~-0.78) | <0.001 | -3.12 (-4.08~-2.16) | <0.001 | -0.52 (-0.78~0.25) | 0.134 |
| **Age (1-year increase)** | 0.12 (-0.20~0.26) | 0.095 | 0.47 (0.13~0.81) | 0.006 | 0.32 (0.22~0.41) | <0.001 |
| **Living arrangement** (Ref: Living with both parents) |  |  |  |  |  |  |
| Living with a single parent | 1.17 (0.45~1.88) | 0.001 | 3.50 (1.77~5.23) | <0.001 | 0.54 (0.11~0.97) | 0.014 |
| Living with others | 0.79 (-0.01~1.58) | 0.053 | 3.18 (1.14~5.22) | 0.002 | 0.54 (-0.06~1.14) | 0.078 |
| **Family relations** (Ref: Good) |  |  |  |  |  |  |
| Average | 2.05 (1.38~2.71) | <0.001 | 7.22 (5.65~8.79) | <0.001 | 1.41 (1.00~1.82) | <0.001 |
| Poor | 4.42 (3.05~5.79) | <0.001 | 12.99 (9.86~16.12) | <0.001 | 2.50 (1.65~3.35) | <0.001 |
| **Student-teacher relations** (Ref: Good) |  |  |  |  |  |  |
| Average | 1.78 (1.16~2.39) | <0.001 | 6.88 (5.44~8.32) | <0.001 | 1.11 (0.74~1.48) | 0.003 |
| Poor | 3.98 (1.41~6.56) | 0.002 | 8.65 (3.73~13.57) | 0.001 | 1.93 (0.65~3.22) | <0.001 |
| **Ever smoking** (Ref: No) | 1.63 (-0.35~3.61) | 0.107 | 5.41 (1.16~9.65) | 0.013 | 1.70 (0.61~2.80) | 0.002 |
| **Ever drinking** (Ref. No) | 1.53 (1.08~1.98) | <0.001 | 4.42 (3.34~5.49) | <0.001 | 1.07 (0.78~1.36) | <0.001 |

Abbreviations: 95% CI, 95% confidence interval; Ref., reference.

*: Unadjusted generalized linear mixed models.

| **Supplementary Table 3. Factors associated with anxiety symptoms, depressive symptoms, and poor sleep quality at Wave 3.** | | | | | | |
| --- | --- | --- | --- | --- | --- | --- |
|  | **Wave 3 (n=1790)^*^** | | | | | |
|  | **Anxiety symptoms** | ***P*** | **Depressive symptoms** | ***P*** | **Poor sleep quality** | ***P*** |
|  | **Unstandardized *β* (95% *CI*)** |  | **Unstandardized *β* (95% *CI*)** |  | **Unstandardized *β* (95% *CI*)** |  |
| **Sex** (Ref: Girls) |  |  |  |  |  |  |
| Boys | -1.25 (-1.67~-0.83) | <0.001 | -2.88 (-3.91~-1.85) | <0.001 | -0.75 (-1.05~-0.45) | <0.001 |
| **Age (1-year increase)** | 0.14 (-0.40~0.32) | 0.127 | 0.45 (-0.04~0.94) | 0.069 | 0.16 (0.06~0.25) | 0.001 |
| **Living arrangement** (Ref: Living with both parents) |  |  |  |  |  |  |
| Living with a single parent | 0.87 (0.13~1.60) | 0.021 | 2.43 (0.58~4.28) | 0.010 | 0.48 (-0.05~1.01) | 0.078 |
| Living with others | -0.08 (-0.83~0.68) | 0.847 | 1.99 (-0.21~4.18) | 0.076 | 0.38 (-0.30~1.05) | 0.275 |
| **Family relations** (Ref: Good) |  |  |  |  |  |  |
| Average | 1.94 (1.20~2.69) | <0.001 | 6.91 (5.26~8.55) | <0.001 | 1.31 (0.80~1.82) | <0.001 |
| Poor | 5.41 (3.86~6.97) | <0.001 | 16.39 (13.37~19.42) | <0.001 | 2.76 (1.68~3.83) | <0.001 |
| **Student-teacher relations** (Ref: Good) |  |  |  |  |  |  |
| Average | 1.47 (0.86~2.09) | <0.001 | 1.47 (0.86~2.09) | <0.001 | 1.05 (0.65~1.44) | <0.001 |
| Poor | 0.71 (-1.57~2.98) | 0.541 | 0.71 (-1.57~2.98) | 0.541 | 0.16 (-1.63~1.94) | 0.862 |
| **Ever smoking** (Ref: No) | 1.35 (-0.01~2.71) | 0.051 | 3.47 (0.18~6.76) | 0.039 | 1.08 (0.15~2.00) | 0.023 |
| **Ever drinking** (Ref. No) | 1.62 (1.15~2.10) | <0.001 | 4.03 (2.88~5.18) | <0.001 | 1.22 (0.89~1.55) | <0.001 |

Abbreviations: 95% CI, 95% confidence interval; Ref., reference.

*: Unadjusted generalized linear mixed models.
